# Artificial intelligence in cardiovascular medicine: prevention, diagnosis, and intervention

**DOI:** 10.3389/frai.2026.1789718

**Published:** 2026-05-18

**Authors:** Sarab Anand, Marco Tagliafierro, Ali Fatehi Hassanabad, Marco Pirelli, Luigi Pirelli

**Affiliations:** Department of Surgery, Laboratory Division of Cardiothoracic Surgery, Columbia University Irving Medical Center, New York, NY, United States

**Keywords:** AI, ECG, echocardiography, SAVR, TAVR

## Abstract

Recent evidence in the literature suggests that Artificial intelligence (AI) is rapidly becoming more clinically relevant with expanding applications across cardiovascular medicine and cardiothoracic surgery. Advances in computational power and the widespread digitization of clinical data have enabled AI models to identify complex, nonlinear patterns across multimodal datasets, positioning them as powerful tools for diagnosis, risk stratification, and procedural decision support. This review examines the current and emerging landscape of AI in cardiac care, with a particular focus on valvular heart disease. We synthesize evidence spanning diagnostic applications such as electrocardiographic and echocardiographic interpretation, preoperative planning, and risk prediction for surgical and transcatheter interventions, and real-time intraoperative decision support. Across these domains, AI systems frequently demonstrate performance comparable to or exceeding conventional approaches, particularly in automating standardized tasks and enabling personalized risk assessment. However, most evidence to date derives from retrospective studies, and challenges related to generalizability hold significant barriers to widespread adoption. We further discuss ethical considerations necessary for safe and equitable implementation. Overall, AI shows substantial promise to augment cardiovascular care across the continuum of practice, but its successful translation into routine clinical use will require rigorous prospective validation, transparent model development and interpretability, and carefully designed integration into existing clinical workflows.

## 1 Introduction

Artificial intelligence (AI) has expanded rapidly within healthcare since its early development in the 1960s, with its role accelerating in recent years alongside greater computational power and the increasing availability of digital clinical data (Gupta et al., 2022). Its capacity to detect complex, non-linear patterns across large, multimodal datasets makes it particularly well-suited for medicine, where laboratory values, imaging, clinical histories, biometrics, and procedural variables often intersect to inform clinical judgment.

AI is rapidly being integrated into cardiovascular medicine and cardiothoracic surgery. While early work focused on automating routine and standardized analysis tasks, more recent advances demonstrate promising applications across the full continuum of care, ranging from population-wide screening and monitoring and pre-intervention clinical decision making to intraoperative guidance and postoperative management (Gupta et al., 2022). As heart disease remains a leading cause of morbidity worldwide, AI has the potential to improve long-term outcomes with integration at every stage of medical care (Dattani et al., 2023).

**Figure 1:**
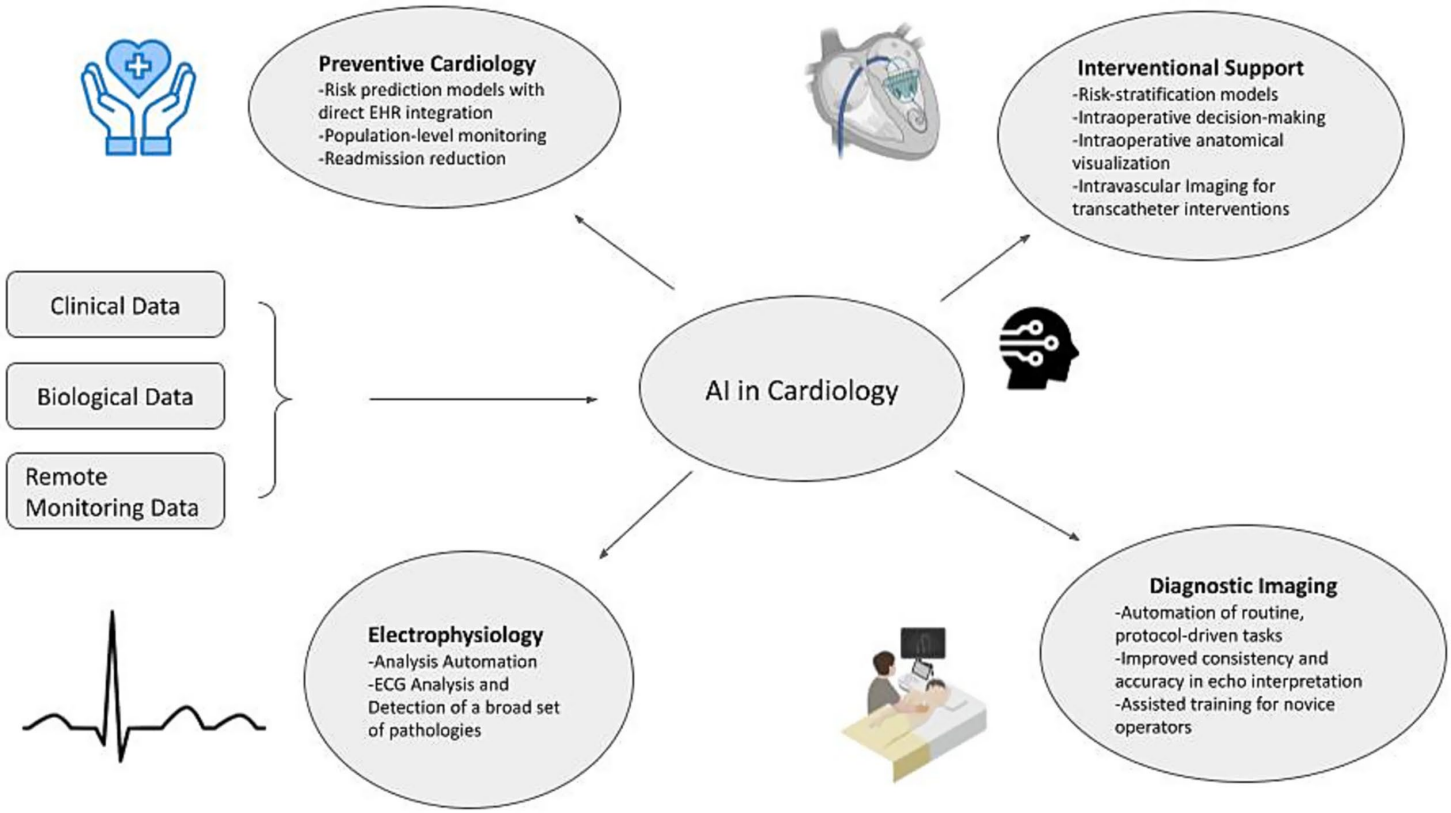
Diagram representing applications of AI within cardiology. Created with the help of Biorender.com.

This review examines the current and emerging landscape of AI within cardiac medicine and cardiothoracic surgery, with an emphasis on valvular disease. The scope includes applications in diagnostics, operative decision support, and postoperative monitoring, as well as broader implications for workflow efficiency, health-system adoption, and global access to care. By synthesizing available evidence, this review aims to characterize AI’s present capabilities, identify ongoing limitations and concerns, and outline future directions necessary for safe, effective, and equitable integration into cardiac surgical practice, as summarized by Figure 1.

## 2 A primer on AI-related terms

*Artificial intelligence (AI)*: a broad concept that describes any initiatives that use a computer to mimic human intelligence and cognition (Pettit et al., 2021). Please refer to the definition of machine learning below to clarify how the term AI is used in the context of this review.

*Machine learning (ML)*: a subset of AI that focuses on building systems that improve their performance by learning patterns from data rather than relying solely on explicitly programmed rules. In most traditional ML approaches, humans transform raw data into more meaningful and relevant inputs, called features, which capture important aspects of the data (IBM, 2026). The model then learns how to map those features to outputs. This means the model’s performance often depends on how well those features are chosen and constructed. The learning process involves optimizing the model based on errors made on training, enabling it to make predictions or decisions on new, unseen data (Pettit et al., 2021). Although machine learning is a subset of artificial intelligence, all AI applications discussed in this review are ML-based; accordingly, the terms AI and ML are used interchangeably. This usage aligns with current literature in the field, which defines AI in practice as systems that learn from data to generate accurate predictions on new, unseen inputs (Pettit et al., 2021).

*Deep learning (DL)*: a subset of machine learning that uses algorithms with layered structures, called neural networks, which are loosely inspired by the human brain. These networks contain many layers (hence, “deep”) and learn complex patterns directly from raw data by processing it through successive transformations. Early layers detect simple patterns in the data, while later layers combine these into more abstract and meaningful representations. Because of this hierarchical structure, deep learning models automatically discover the most useful features during training, reducing the need for human pre-processing. This makes deep learning particularly powerful for unstructured and complex data such as images, audio, and text, as well as for tasks that involve connecting multiple data types (Pettit et al., 2021).

## 3 History of AI within cardiac medicine

Cardiovascular diseases have long been among the most prevalent health challenges worldwide (Dattani et al., 2023; Taheri Soodejani, 2024). Given the volume of routinely collected clinical data and the standardized diagnostic testing embedded in cardiac care, cardiology has become a focal point for the integration of artificial intelligence in medicine.

### 3.1 AI in electrocardiogram analysis

The application of artificial intelligence (AI) in cardiology began in the 1990s, when early models were explored as experimental alternatives to rule-based electrocardiogram (ECG) interpretation software. The application of artificial intelligence (AI) in cardiology began in the 1990s, when early models were explored as experimental alternatives to the rule-based electrocardiogram (ECG) interpretation software (Haq et al., 2022; Macfarlane and Kennedy, 2021). Traditional ECG algorithms used Boolean logic and decision trees to infer certain pathologies based on clinical guidelines (e.g., QRS duration of more than 0.10 s but less than 0.12 s, Absence of Q Wave in V5, V6, I lead, etc.) (Willems et al., 1985). This inflexible structure makes them prone to mischaracterizations in subthreshold and atypical presentations. For example, the mischaracterization of an atypical right bundle branch block as a ST-segment elevation myocardial infarction, due to difficulties in delineating the QRS complex from ST segments, has been a common issue in ECG interpretation software (Littmann, 2023).

Since the early 2000s, AI-based ECG interpretation systems have advanced substantially in performance and in clinical relevance. Modern DL models now routinely exceed human diagnostic accuracy while operating at significantly faster speeds (Oke and Cavus, 2025). For example, in one retrospective case, a convolutional neural network developed from a data of 180,000 ECGs from over 70,000 patients demonstrated an exact diagnostic match in 80% of test cases, surpassing average physician performance across experience levels (Zhu et al., 2020).

More importantly, AI ECG systems are expanding the functional role of the ECG beyond traditional pattern recognition. Because these models are trained on large datasets incorporating clinically relevant patient metadata, they can identify patterns that are undetectable to human reviewers. Multiple retrospective studies and meta-analyses now demonstrate that DL models can detect a wide range of cardiovascular pathology, including left ventricular systolic dysfunction (LVSD), silent atrial fibrillation (AF), hypertrophic cardiomyopathy (HCM), and even systemic biomarkers, metabolic bioproducts, and electrolytes such as serum potassium levels (Attia et al., 2021; Elias et al., 2024). Models have also shown strong discriminatory performance for valvular heart disease, including moderate-to-severe aortic stenosis (AS) and mitral regurgitation (MR), across diverse populations (Cohen-Shelly et al., 2021; Kwon et al., 2020).

The combination of these diagnostic capabilities with ECG’s ubiquity positions AI ECG programs as a powerful tool for rapid phenotyping, population-level screening, and preventive cardiology. The increasing availability of wearable and at-home ECG monitoring systems further supports use cases such as early detection, risk stratification, and remote monitoring for high-risk individuals (Lee et al., 2022).

Emerging evidence suggests these tools may also identify disease trajectories before clinical onset. For instance, algorithms that classify normal-appearing ECGs as abnormal have been shown to predict a five-fold increased likelihood of developing LVSD within 5 years. These findings suggest the potential possibility of a “previvor” category representing patients at elevated, quantifiable risk (Attia et al., 2021). Similarly, models capable of detecting paroxysmal AF from sinus rhythm or early signatures of HCM highlight how AI-ECG may support earlier intervention, targeted diagnostics, and prevention-based care pathways (Siontis et al., 2021).

It is critical to note that the literature mentioned so far has all been retrospective. Still, there is a growing body of prospective literature. For example, a prospective, non-randomized interventional study showed that AI-guided targeted screening significantly increased the detection of previously undiagnosed atrial fibrillation compared to usual care (Noseworthy et al., 2022). Similarly, the FDA has currently approved multiple AI-ECG products for AF and low LVEF, among several more for wearable/at-home devices for arrhythmias (Palermi et al., 2025).

### 3.2 AI in echocardiographic analysis

Starting in the 2010s, AI and ML techniques started to be applied for the analysis of echocardiograms. Echocardiograms remain the first-line for diagnostic and prognostic analysis of a significant portion of cardiac diseases (Schuuring et al., 2021). Yet, at the same time, their overall processing workflow remains tedious, requiring, at best, the complex coordination between a network of cardiologists and technicians in larger hospital environments for the management of such a high workload of analyses, and, at worst, requires cardiologists in smaller-practice settings to spend significant time conducting routine, standardized aspects that could be allocated to more cognitively demanding efforts. Perhaps most importantly, echocardiography readings often require human interpretation, meaning they are prone to inter-subject variability (Gupta et al., 2022; Alsharqi et al., 2018).

AI has the potential to improve workflows and standardization. Current AI echocardiogram systems have already proven high efficacy in the accurate measurement of anatomical measures of the heart like chambers or annulus measurements (Alsharqi et al., 2018; Barry et al., 2023). DL techniques and neural networks, in particular, are well-suited for echocardiography because they can learn complex, hierarchical image features directly from data, allowing them to robustly identify cardiac structures despite noise, artifacts, variability in imaging and anatomies (Krittanawong et al., 2023).

Current literature shows AI’s growing progression within the echocardiography space, moving beyond basic task automation and towards a more integrated and cohesive diagnostic output through the use of multiple clinically relevant variables. For example, one study used several machine learning techniques to create a model capable of differentiating pathological conditions that share similar phenotypes. The model was able to distinguish non-clinically relevant left ventricular hypertrophy, which is often seen in athletic populations, from its clinically serious counterpart in hypertrophic cardiomyopathy by incorporating variables such as age (Alsharqi et al., 2018). Other studies within the same literature review have further emphasized that machine learning models can accurately differentiate between pathologies with similar presentations, where no single diagnostic parameter currently exists to make a reliable distinction (Alsharqi et al., 2018). These findings highlight the possibility that AI may eventually outperform human operators and detect subtleties that are not readily apparent through standard interpretation (Alsharqi et al., 2018).

Ultimately, this example reflects AI’s ability to contextualize data using a large body of training information, which exceeds what is possible with traditional algorithmic or rule-based approaches.

## 4 AI for pre-operative planning in valvular and cardiac operations

Artificial intelligence has the ability to enhance the precision, efficiency, and personalization of cardiac surgical care. Across cardiovascular operations, AI is being used to improve preoperative planning by enhancing surgical metric workflows and providing context and nuance to support physicians’ clinical decision-making.

### 4.1 Surgical parameter acquisitions

AI has been increasingly integrated to automate preoperative planning processes, particularly in imaging, such as computer tomography (CT) and magnetic resonance imaging (MRI) images, to improve processes like segmentation, measurement extraction, and 3-dimensional reconstruction (Mank et al., 2025). Improving accuracy and reducing the time needed for visualization of anatomical structures is critical for surgical decision-making (Mank et al., 2025; Toggweiler et al., 2024). Several studies have created learning models that measure several surgical parameters, such as annular measurements, in a fraction of the time it would take a senior observer and with measurement accuracies ranging from comparable to superior (Mank et al., 2025; Toggweiler et al., 2024). These tools will ultimately streamline pre-operative workflows, enabling surgical teams to move from planning to intervention more efficiently.

### 4.2 Risk stratification and outcome prediction: AI versus conventional risk scores

One rapidly advancing area of AI integration with cardiovascular operative planning is pre-intervention risk stratification and outcome prediction. Traditional risk calculators, most notably, the Society of Thoracic Surgeons (STS) and EuroSCORE II calculators, estimate perioperative outcomes, namely 30-day mortality, using a preset of demographic, clinical, and laboratory values (EuroScore, 2025; STS ACSD, 2025). However, multiple studies have demonstrated that machine learning models frequently outperform both STS and EuroSCORE II in predicting perioperative mortality across surgical and transcatheter procedures (Fan et al., 2022; Gomes et al., 2021; Sulague et al., 2024). For example, retrospective studies of elective cardiac surgery cases have reported that learning models were both more accurate at predicting mortality than the Euroscore II calculator and provided greater clinical utility (Allou et al., 2023; Allyn et al., 2017).

These findings have held largely consistent in studies examining aortic valvular interventions, specifically. One retrospective study by Kilic et al., for example, demonstrated “excellent calibration and modest improvements in discriminatory ability” in comparison to existing STS models for a cohort of patients undergoing isolated surgical aortic valve replacement (SAVR) (Kilic et al., 2021; Kilic et al., 2021).

Similar results have been found in transcatheter aortic valve replacement (TAVR) specific studies with learning models showing improved accuracy in predicting in-hospital mortality risk compared to a TAVR-specific STS calculator as well as the Euroscore II calculator (Sazzad et al., 2024; Mohammadyari et al., 2024; Hernandez-Suarez et al., 2019).

AI models’ ability to identify clinically meaningful patterns within large datasets makes them well-positioned to accelerate personalized risk prediction through the integration of biomarkers. Growing evidence supports the utility of readily available cardiac biomarkers, such as high-sensitivity troponin T (hsTnT) and N-terminal pro-brain natriuretic peptide (NT-proBNP), in guiding treatment pathways for patients with severe AS, yet these variables remain absent from current risk stratification tools (Barbieri et al., 2024).

In response to this gap, Barbieri et al. (2024) developed the ABC-AS neural-network-based score, incorporating age, hsTnT, and prior cardiac decompensation. The model outperformed traditional tools like STS in predicting cardiovascular mortality and demonstrated consistent predictive value across both TAVR and SAVR cohorts.

One reason that ML models might provide more accurate and individualized risk prediction compared to traditional calculators is that they are not constrained by variable input capacity; existing tools are constrained by the limited variables they allow users to input, which in turn restricts their diagnostic applicability and full clinical utility. For example, the current app version (2.0.6) of the STS Adult Cardiac Surgery Operative Risk Calculator does not allow clinicians to specify prior TAVR (EuroScore, 2025; STS ACSD, 2025). Omission of this clinically significant factor likely underestimates predicted morbidity, mortality, and overall procedural risks. Such examples highlight AI models’ flexibility.

### 4.3 Secondary outcomes and complication prediction

AI’s role in cardiac intervention planning extends beyond just mortality prediction, but to forecasting secondary outcomes, which play a crucial role for patients with complex medical histories and multiple comorbidities. Recently developed models have started to predict complications such as acute kidney injury and prolonged postoperative ventilation after cardiovascular intervention, offering clinician’s earlier insight into potential risks. By expanding predictive focus beyond traditional endpoints, these tools will aid in supporting a more holistic approach to patient care and risk assessment (Shojaei et al., 2025).

### 4.4 Clinical and practical implications

In summary, AI-driven risk stratification is likely to be a highly clinically relevant tool in the future. By leveraging multimodal data and advanced learning methods, AI has repeatedly been proven in the retrospective literature to outperform conventional scores in predicting mortality and postoperative complications across both surgical and transcatheter valve interventions. The next phase will require rigorous validation, including multicenter prospective and randomized trials, to ensure generalizability across diverse patient populations. Model interpretability will be critical to support trust and safe deployment. Integration into clinical practice will also require careful ethical and implementation frameworks, including equitable benefit, outcome evaluation, and scalability across diverse care settings. Together, these steps will be necessary to transition AI from promising prototypes to trusted clinical tools. Further discussion of these frameworks is provided in the Discussion section.

## 5 AI integration within electronic health records (EHRs)

AI’s capacity to identify nonlinear patterns and subtle associations across large datasets has expanded its role in pre-intervention planning and risk stratification. As models continue to evolve toward more personalized prediction, their effectiveness will depend on access to increasingly large and diverse data sources. The ongoing digitization of healthcare, particularly through electronic health records (EHRs), positions EHR systems as a natural environment for AI deployment. These platforms store extensive structured data (e.g., labs, vitals, medications) and unstructured data (e.g., clinical notes, imaging reports), offering a rich foundation for predictive analytics and real-time clinical decision support.

### 5.1 Current applications and emerging directions

Although AI integration within EHRs for cardiovascular applications remains early, existing work demonstrates clear potential. For example, an EHR-embedded model outperformed guideline-recommended tools in identifying low-risk patients with gastrointestinal bleeding in the emergency department setting (Shung et al., 2024). In cardiac surgery, comparable model-developed scores could support risk differentiation between valve replacement strategies, anticipate post-operative complications, or inform procedural timing as discussed in detail earlier.

Adoption of risk scores in EHR’s is already underway. Epic Systems®, which holds 41% of the U.S. hospital EHR market share, for example, has worked to integrate risk scores for hospital systems based on the success of their clinical trials, validating their measures (STAT, 2026; Varley et al., 2021). When the University of Pittsburgh Medical Center, for example, wanted to integrate a measure that had previously been shown to reduce the risk of 1-year mortality after surgical intervention for frail adults by nearly 3-fold after implementation, they worked with Epic to integrate it into their EHR and saw a rise in care-team compliance from 58 to 84% (Varley et al., 2021).

One notable, ongoing trial is investigating the utility of EHR-integration into structural heart condition management. The Addressing undertreatment and heaLth Equity in aortic stenosis and mitral regurgitation using an integrated ehR plaTform (ALERT) trial is a “multicenter, prospective, cluster-randomized controlled trial, designed to evaluate the impact of automated electronic health record notifications on the management of severe AS and MR” (Batchelor et al., 2026). Likely, we will likely see similar trials in the future investigating the efficacy of AI-developed risk scores. Similarly, these trials will have to show AI-developed risk scores provide greater accuracy than traditional risk scores to warrant adoption. One reason is that ‘in-house’ models—trained on patient data from the health system in which they would be deployed—may provide more accurate predictions than traditional risk scores derived from broader cohorts (Garg et al., 2025). However, this is a double-edged sword, as such models may also reinforce existing biases within that system’s clinical practices (Göçer and Barış Durukan, 2025; Binkley et al., 2022).

Advances in natural language processing (NLP) offer another dimension to the role that AI will play in EHR’s. NLP methods enable the extraction of clinically relevant information from narrative text, converting previously inaccessible unstructured notes into usable input for deep learning models (Juhn and Liu, 2020; Kaswan et al., 2021; Murff et al., 2011). At scale, newer system designs have demonstrated the ability to process tens of thousands of EHR updates daily and deliver AI-derived insights across multispecialty environments (Suryanarayanan et al., 2021). In line with this broader trend, Northwestern Med has partnered with Tempus AI, an AI health technology company, to integrate its AI tools into the electronic health record (Carron, 2026). In a Tempus case study, they leveraged a natural language processing model to help clinicians at a large academic medical center identify patients with severe aortic stenosis and mitral regurgitation who “may have been previously overlooked” (Luong, 2026).

Beyond predictive accuracy, AI may strengthen clinical decision-making by advancing current clinical decision support systems (CDSS). Traditional CDSS rely on static rules, alerts, and guideline-based logic, often resulting in limited contextual relevance and alert fatigue. AI-enabled systems offer the potential for adaptive, patient-specific, and context-aware recommendations, representing a meaningful evolution in how clinicians might interact with AI during care delivery (Thamma, 2025).

## 6 AI within the intraoperative setting

AI is increasingly being explored for intraoperative, real-time decision-making support in cardiac surgery.

One way that AI is being integrated into decision-making support is through real-time processing. During TAVR’s, models can analyze invasive pressure tracings in real time to detect valve malposition or hemodynamic instability, allowing for immediate corrective action. Patient-specific computational modeling is also being used to predict complications and inform adjustments during unexpected anatomical scenarios. Looking ahead, AI-integrated augmented reality may enhance intraoperative visualization by overlaying real-time, context-aware guidance onto the surgical field, supporting more precise and efficient decision-making. (Leivaditis et al., 2025; Maznyczka et al., 2025; Shin et al., 2025).

In open-chest congenital procedures, video-based machine learning has shown promise for guidance, using motion analysis of the beating heart to predict outcomes before chest closure, which can guide surgeons’ decision-making during critical intraoperative moments (Lo Muzio et al., 2021).

While still early in development, emerging work suggests that AI may soon be capable of synthesizing diverse intraoperative data streams to offer real-time surgical decision-making recommendations. A recent preprint describing CardiacGPT, an AI wrapper using Open-AI and Claude models to generate patient-specific operative guidance, demonstrated that generative models can produce dynamic, context-aware recommendations using multimodal intraoperative data (Ramezani et al., 2025). In a blinded review of 500 cardiac surgery cases, the highest-performing models received over 98% high-trust ratings from attending surgeons, indicating early feasibility and clinician receptiveness to LLM-based real-time guidance in high-risk operative settings (Ramezani et al., 2025).

AI is well-suited to enhance minimally-invasive and robotic surgery (Wah, 2025). Current literature indicates that AI is already being investigated for integration into these systems in ways consistent with themes discussed throughout this review, including improved automation of data processing and the synthesis of monomodal biometric inputs into more informative, multimodal representations (Wah, 2025). In robotic surgery, such overlays can be delivered directly at the surgeon’s console, supporting more precise intraoperative decision-making (De Backer et al., 2023). Ward et al. highlight the advantages of neural networks for these applications, noting that under the variable visual conditions inherent to surgery (e.g., anatomical differences, smoke, blood, or image blur), they maintain consistent instrument identification, whereas traditional machine learning models would fail (Ward et al., 2021).

Although the literature specifically examining AI-enabled robotic surgery for valvular disease remains limited, early studies of AI-assisted robotic minimally invasive coronary artery bypass grafting report promising results, including shorter operative times, reduced blood loss, and lower complication rates compared with conventional approaches (Rivero-Moreno et al., 2024).

## 7 Ethical implications, considerations, and learning-points

### 7.1 Ethical factors

While artificial intelligence holds promise for advancing cardiovascular care, its integration introduces several risks and ethical challenges that must be addressed before widespread adoption (Göçer and Barış Durukan, 2025; Loftus et al., 2020).

AI models have the potential to improve equitable outcomes for marginalized populations through early prevention screening and reduction in surgical decision-making bias; however, they also have the potential to perpetuate existing disparities (Binkley et al., 2022). AI models rely heavily on the quality and representativeness of their training datasets. Models trained on historical datasets that underrepresent or misrepresent certain populations could produce inaccurate or biased predictions, potentially leading to diagnostic errors or inappropriate treatment decisions, and reinforcing existing disparities in cardiovascular outcomes (Binkley et al., 2022; Akingbola et al., 2024; Hashimoto et al., 2018).

Preventing AI from leading to biased results will require models to be given diverse, representative datasets, relevant, contextual variables such as lifestyle factors and social determinants of health, and ongoing rigorous bias evaluation of algorithms (Göçer and Barış Durukan, 2025; Cross et al., 2024; Johnson-Mann et al., 2021).

Ongoing evaluation of bias in AI outputs might hold a particular challenge, given the “black-box” nature of learning models (Kilic et al., 2021; Göçer and Barış Durukan, 2025; Hashimoto et al., 2018; Cross et al., 2024). This challenge represents a larger issue within AI’s integration into supporting clinical and operative decision-making. The lack of transparency within AI models’ reasoning and decision-making processes presents issues for physicians trying to interpret outputs that differ from their own clinical judgment. Reconciliation of these differences within an intraoperative setting could potentially disrupt operative workflow and team-based coordination, leading to increased risk (Khodaveisi et al., 2025; Whitbread et al., 2025).

In a non-operative setting, differences between human clinical judgement and AI recommendations might lead to wasted time, medical bloat, and potentially hinder a physician’s ability to communicate decision rationales to their patients, potentially eroding doctor-patient communication and trust (Göçer and Barış Durukan, 2025; Adegbesan et al., 2024; Akingbola et al., 2024; Petch et al., 2022).

These issues raise a larger ethical concern of where responsibility lies within AI-supported decision-making; if an AI-supported recommendation results in harm, determining whether responsibility lies with the physician, the developer or the institution remains unclear (Göçer and Barış Durukan, 2025; Loftus et al., 2020; Sarofim, 2024).

Problems with AI are not limited to disagreements with AI outputs and human judgment processing. AI’s growing accuracy and efficiency also pose challenges for clinical skill retention. As automated systems take on increasingly complex interpretive tasks from measurement (ECG/imaging) analysis to even intra-operative decision making, clinicians risk becoming overdependent on these tools. This “de-skilling” could erode physicians’ baseline interpretive abilities and independent clinical judgment (Göçer and Barış Durukan, 2025; Loftus et al., 2020; Adegbesan et al., 2024; Sarofim, 2024). AI, however, has shown promise in actually improving a clinician’s skillset. Several articles in the literature have noted that AI could play a pivotal role in training novice echocardiogram readers, given the steep learning curve (Gupta et al., 2022; Schuuring et al., 2021).

Together, these considerations underscore that the successful integration of AI in cardiovascular care will require careful governance, transparent model development, and ongoing oversight. Ensuring that AI strengthens, rather than undermines, equitable and clinically sound decision-making will be essential as these systems transition from experimental use to routine practice.

### 7.2 Safety frameworks for research, implementation, and deployment

Ethical and equitable integration of AI into cardiovascular medicine requires established clinical safety and validation frameworks to guide its development and deployment. Standardized reporting guidelines such as CONSORT-AI and SPIRIT-AI have helped formalize how AI-driven clinical studies are designed and reported, promoting transparency, reproducibility, and methodological rigor (Liu et al., 2020). At the same time, domain-specific frameworks are necessary to account for the unique challenges of cardiovascular applications. For example, the PRIME 2.0 checklist provides a comprehensive structure for evaluating cardiovascular imaging AI across the full research lifecycle, from data preprocessing and model development to clinical validation and reporting, while also addressing emerging complexities such as multimodal data and dynamic cardiac physiology (Kagiyama et al., 2026).

Beyond study design, effective implementation requires frameworks that extend into real-world clinical use. The American Heart Association’s 2025 scientific advisory proposes a pragmatic, risk-based approach spanning pre-deployment validation, workflow-integrated implementation, and continuous post-deployment monitoring. This emphasizes that AI systems must not only demonstrate initial validity, but also maintain performance, safety, and equity across diverse clinical settings over time (Jain et al., 2025). Underlying these technical frameworks is the need to align AI use with core principles of medical ethics, including autonomy, beneficence, nonmaleficence, and justice. In this context, organizations such as the American Medical Informatics Association have highlighted the importance of governance structures that prioritize transparency, accountability, and explainability, particularly in addressing concerns surrounding bias, data privacy, and the “black-box” nature of many AI models (Solomonides et al., 2021). Together, these frameworks emphasize that AI tools will require continuous evaluation and monitoring to ensure they meet standards of safety, reliability, and ethical responsibility.

## 8 Discussion

**Figure 2:**
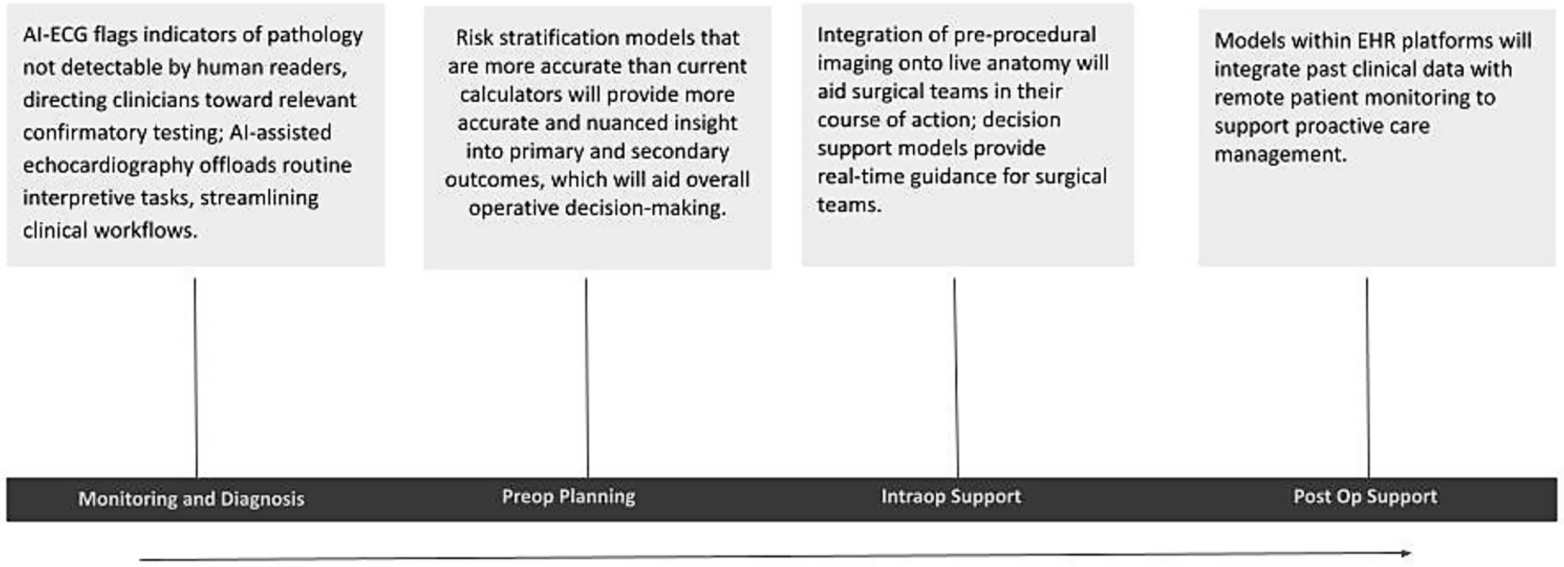
Role of AI in cardiology across the continuum of care.

As artificial intelligence research continues to expand, so too do its applications across clinical cardiovascular medicine and cardiothoracic surgery. The evidence reviewed demonstrates meaningful advancement across the continuum of care, including diagnostic support, preoperative planning, intraoperative guidance, and postoperative monitoring, as illustrated in Figure 2. AI shows particularly strong performance in automation, multimodal data pattern recognition, and the reduction of human error. However, it is still important to recognize that these findings are almost exclusively studied in retrospective cohorts; whether their validity and generalizability will translate to a prospective setting remains to be determined.

Collectively, these developments reflect how AI contributes to clinical reasoning across different “grades” of cognitive similarity to human thinking, as referenced in Table 1. Grade I AI enables clinicians to focus on higher-order elements of care through automation of technically complex, but standardized processes, such as ECG interpretation or surgical parameter extraction. Grade II, AI models provide enriched cognitive support through nuanced risk differentiation and personalized outcome offering clinicians context beyond what current risk scores or rule-based decision support systems are capable of. At the most advanced end of the spectrum, AI is evolving toward independent synthesis—integrating multimodal datasets to identify patients who are likely to require future cardiovascular intervention and inform clinical and operative decision-making. While Grade I and II models are actively being tested prospectively for screening, detection, and care-planning workflows, it is likely in the future that Grade III models may potentially be involved across the continuum of cardiovascular care.

**Table 1:** Grades of AI contribution to clinical workflows and decision support.

Grade	Intended contribution	Description
Grade I: task automation	Workflow efficiency	AI automates routine, protocolized tasks (e.g., image segmentation, measurement extraction, report pre-population), reducing clinician workload and allowing greater focus on higher-level clinical reasoning.
Grade II: clinical augmentation	Enhanced interpretation & risk assessment	AI provides more accurate, granular, and context-aware insights than conventional metrics which informs clinical decision-making.
Grade III: decision support	Predictive and prescriptive guidance	AI integrates multimodal data to generate evidence-based recommendations, such as predicting treatment outcomes, suggesting diagnostic pathways, or identifying patients at risk for deterioration or readmission, while clinicians retain final authority.

This progression underscores the need for deliberate consideration of AI’s role in medicine, ensuring that ethical frameworks, clinical expectations, and governance structures evolve alongside technological capability rather than react to it retrospectively. Successful implementation will depend not only on continued technical refinement but also on the establishment of robust oversight mechanisms, transparent validation standards, and scalable regulatory pathways. As outlined earlier, challenges related to interpretability, accountability, equity, and potential clinician skill erosion must be addressed proactively—particularly in high-risk environments such as cardiac surgery, where decisions are made dynamically and may directly affect morbidity and mortality.

Overall, the current body of evidence points toward a cautious yet optimistic trajectory. AI has demonstrated substantial potential to augment cardiovascular care, but realizing this promise will require rigorous prospective evaluation, multi-center collaboration, seamless EHR integration, and regulatory guidance designed for adaptive learning systems.

## 9 Conclusion

Artificial intelligence in cardiovascular medicine is best understood as a continuum, ranging from task automation to integrated decision support that reshapes the between clinicians and data-driven decision-making. Currently, AI has shown strong potential to support clinicians through improved automation and precision; however, its prospective validation and generalizability remain to be established. As AI’s role in care deployment becomes higher-order, the more emphasis is required on careful oversight. Robust prospective validation, transparent model development, and standardized regulatory frameworks will be essential to ensure safety, reliability, and accountability. Equally important is the need for continuous monitoring to mitigate bias and ensure equitable deployment. Ultimately, the responsible integration of AI will determine whether its promise translates into meaningful, patient-centered improvements in care.
